# Under-Five Mortality and Associated Risk Factors in Children Hospitalized at David Bernardino Pediatric Hospital (DBPH), Angola: A Hierarchical Approach

**DOI:** 10.3390/ijerph21081062

**Published:** 2024-08-14

**Authors:** Israel C. Avelino, Joaquim Van-Dúnem, Luís Varandas

**Affiliations:** 1 Global Health and Tropical Medicine (GHTM), LA-REAL, Instituto de Higiene e Medicina Tropical (IHMT), Universidade Nova de Lisboa, 1169-056 Lisboa, Portugal; 2 Clínica Multiperfil, Luanda 2177, Angola; 3 Faculty of Medicine, Agostinho Neto University, Luanda 815, Angola; joaquim.vandunen@uan.ao; 4 Nova Medical School, Faculdade de Ciências Médicas, Universidade Nova de Lisboa, 1169-056 Lisboa, Portugal; 5 Departamento de Pediatria, Hospital Dona Estefânia, 1169-045 Lisboa, Portugal

**Keywords:** under-five mortality, Angola, determinants of health, child healthcare, case-control study

## Abstract

Reducing under-five mortality is a crucial indicator of overall development in a country. However, in Angola, understanding the factors contributing to hospital deaths in this vulnerable demographic remains incomplete despite improvements in healthcare infrastructure and public health policies. With one of the highest under-five mortality rates in sub-Saharan Africa, Angola faces significant challenges such as malaria, malnutrition, pneumonia, neonatal conditions, and intestinal infectious diseases, which are the leading causes of death among children. This study aimed to identify factors associated with hospital deaths among children aged 28 days to five years admitted to DBPH in Luanda between May 2022 and June 2023. Using a hospital-based case-control design, the study included 1020 children, among whom 340 experienced hospital deaths. Distal and intermediate determinants emerged as primary predictors of hospital mortality, showing significant associations with: mother without schooling (OR [95%CI] 4.3 [1.2–15.7], *p* < 0.027); frequent alcohol consumption during pregnancy (OR [95%CI] 3.8 [2.5–5.9], *p* < 0.001); hospital stay ≤24 h (OR [95%CI] 13.8 [6.2–30.8], *p* < 0.001); poor nutritional status (OR [95%CI] 2.1 [1.4–3.2], *p* < 0.001); short interbirth interval (OR [95%CI] 1.7 [1.1–2.5], *p* < 0.014); maternal age ≤19 years (OR [95%CI] 5.6 [3.0–10.8], *p* < 0.001); and maternal age ≥35 years (OR [95%CI] 2.1 [1.2–3.7], *p* < 0.006). These findings highlight the preventable nature of most under-five hospital deaths and underscore the urgent need to address social inequities and improve the quality of primary healthcare services to effectively reduce child mortality in Angola.

## 1. Introduction

The under-five mortality rate is a crucial indicator of a country’s overall development, reflecting the effectiveness of healthcare services and the socio-economic factors influencing child survival [1,2]. Embedded within the Sustainable Development Goals (SDGs), it remains a critical measure of global progress towards equitable healthcare [3]. 

Significant global strides have been made, with the under-five mortality rate plummeting by 60% from 1990 to 2020. However, regional disparities persist starkly. Sub-Saharan Africa, despite notable improvements, continues to shoulder a disproportionate burden, accounting for nearly half of all global under-five deaths [1]. 

Among the leading causes of hospital mortality in children under five worldwide are complications from premature birth, birth asphyxia/trauma, pneumonia, diarrhea, and malaria [1,4]. Socioeconomic factors such as place of residence, maternal education level, and household wealth, as well as maternal health status and limited access to healthcare, are strongly associated with the risk of under-five mortality in sub-Saharan Africa, a region responsible for nearly 50% of child mortality, despite comprising only 11% of the world’s population [4,5].

Angola ranks among the seven countries in sub-Saharan Africa with the highest under-five mortality rates [2,5], collectively responsible for approximately one-fifth of global under-five deaths [2]. By 2020, Angola had made significant strides, reducing its under-five mortality rate to 71.5 per 1000 live births from 105 in 2012. About 60% of these deaths occur within the first year of life, with over half (54.5%) specifically during the neonatal period [6]. Despite these advancements, Angola remains far away from achieving the Sustainable Development Goals (SDGs) target of 25 under-five deaths per 1000 live births by 2030 [3].

The country has made notable improvements in healthcare infrastructure and public health policies, particularly focusing on maternal, child, and family health [7]. However, challenges persist in reducing child mortality rates [6,8]. Supported by the UN, Angola has been actively working towards implementing the SDGs and the African Union’s Agenda 2063, as evidenced by its first Voluntary National Review (VNR) in 2021 [7].

Nevertheless, gaps remain in documenting hospital mortality risk factors among children under five in Angola. Despite advancements, children under five disproportionately suffer from preventable diseases such as malaria, malnutrition, pneumonia, neonatal conditions, meningitis, intestinal infectious diseases, and acute respiratory infections, which are leading causes of hospitalization and mortality in this age group [9,10]. Understanding these specific factors is crucial for developing effective public health strategies and improving child healthcare in Angola.

This study addresses the urgent need to comprehensively understand the determinants of child mortality in Angola. By systematically examining risk factors from clinical conditions to socioeconomic factors and access to health services, it fills a significant gap in the literature, which predominantly focuses on clinical and epidemiological aspects with limited hierarchical analysis of underlying risk factors [8,9,10]. This nuanced understanding is crucial for informing the development of more effective maternal and child health policies and interventions tailored to local contexts [6].

Moreover, this study’s significance is multifaceted. Theoretically, it expands our understanding of the complex mechanisms contributing to high mortality rates among children under five, enriching existing conceptual frameworks [1,6,11,12,13,14,15]. Practically, the findings directly inform the formulation of public policies and intervention programs capable of addressing both the clinical aspects and social determinants of this challenge. Notably, this study aligns explicitly with the Sustainable Development Goals, underscoring its potential impact on global health agendas.

Several essential theoretical frameworks are pivotal in comprehending and addressing health disparities, particularly concerning mortality among children under five years old in developing countries such as Angola. Intersectionality theory elucidates how social determinants such as race, gender, and socioeconomic status intersect to influence structural inequalities [11]. The Social Determinants of Health (SDOH) framework examines how socio-economic conditions, maternal education, physical environment, and healthcare access impact infant and child mortality [12]. Nancy Krieger’s Ecosocial Theory analyzes interactions among social, environmental, and biological factors in health, with a focus on disparities and environmental dynamics [13]. Equity Theory emphasizes policy interventions aimed at equitably distributing resources and opportunities to foster health equity across diverse population groups [14].

The hierarchical approach proposed by Mosley and Chen is a fundamental analytical tool for understanding under-five mortality, extensively employed in studying determinants of child mortality in developing countries [15]. This conceptual framework delineates three hierarchical levels of determinants influencing child mortality: distal, intermediate, and proximal factors. Distal determinants encompass socioeconomic, cultural, and environmental factors that broadly impact population health and well-being. Intermediate determinants include factors related to healthcare access and utilization, such as availability and quality of healthcare services. Proximal determinants relate directly to individual children and encompass factors such as nutritional status and exposure to infectious diseases [15,16,17].

This hierarchical framework facilitates the assessment of the varying contributions of these factors to the variability in under-five mortality within developing countries. The categorization into distal, intermediate, and proximal levels not only highlights the significance of mortality-associated factors but also organizes them chronologically, aiding in identifying interconnections among determinants and guiding the implementation of targeted corrective and preventive measures.

Therefore, this study aims to identify and analyze determinants of hospital mortality among children from 28 days to five years of age at the David Bernardino Pediatric Hospital (DBPH) in Luanda. Employing a hierarchical approach, this study seeks to provide critical insights for policymakers and healthcare providers to address this urgent issue.

## 2. Materials and Methods

### 2.1. Study Type and Location

A hospital-based case-control study was conducted in a ratio of 1:2 from 1 May 2022 to 30 June 2023. This one-year period allows for the avoidance of potential seasonal bias. This study was carried out in Luanda, at the David Bernardino Pediatric Hospital (DBPH), a tertiary-level hospital that serves as a teaching and research center and is the main reference for pediatric care in the Angolan National Health Service. With 554 inpatient beds and structured into 12 services, the hospital admits an average of 1500 patients per month and has an occupancy rate of 97%. The emergency department, the gateway for hospital admissions, is equipped with 150 observation beds and receives an average of 9000 children per month.

### 2.2. Population and Sample

The study population consisted of children admitted to DBPH during the study period. Cases included children aged between 28 days and five years who died during hospitalization, irrespective of their clinical condition. Controls were children from the same age group who survived during the same period and were selected from patients who met discharge criteria during the same month as the identified cases, matched by age (±6 months) and sex with the corresponding cases. The principal investigator reviewed the final diagnoses and defined the primary diagnosis. Discrepant cases were discussed with the team and decided upon by consensus. The International Classification of Diseases (ICD-10) was used to classify the underlying causes of death.

Cases that died after 4:00 PM and on weekends were automatically excluded due to the inability to interview caregivers. Additionally, cases that died from accidents, those for whom sufficient information could not be obtained, controls with readmissions or prior participation in the study, and cases where the primary caregiver refused to participate were also excluded. The exclusion of traffic accident cases aimed to reduce data heterogeneity, focusing instead on other types of injuries, such as those caused by domestic accidents.

**Figure 1:**
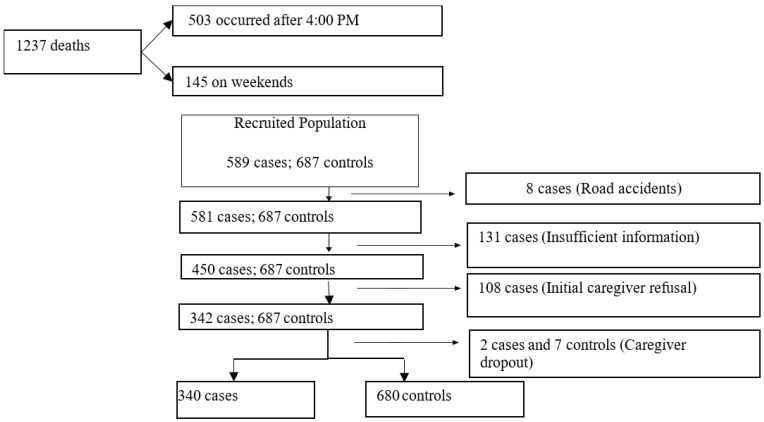
Population selection for the study.

The sample was systematically selected by applying predefined inclusion and exclusion criteria for both cases and controls. Thus, out of the 1237 children who died, 503 deaths occurred after 4:00 PM and 145 occurred on weekends. A total of 589 caregivers were interviewed, of whom 108 refused to participate in the study. A total of 131 cases did not contain sufficient information, 8 were due to road accidents, and two withdrew during the interview, resulting in a response rate of 81.7% (Figure 1).

### 2.3. Procedures and Data Collection Instrument

Data collection was conducted daily, from Monday to Friday, from 8:00 AM to 4:00 PM. Data were obtained through interviews conducted by the researcher with the child’s caregiver, either at admission or within 24 h of hospitalization, using a standardized and coded form containing closed-ended and some open-ended questions regarding sociodemographic aspects, clinical characteristics, and care-related factors (Supplementary File). Additional data were obtained through clinical record review.

### 2.4. Definition and Categorization of Variables

Entry into the labor market: An adaptation of the definition by the Brazilian Institute of Geography and Statistics (IBGE) [18] was used, considering the following categories: (a) employed: engages in economic activity or works in a public or private establishment, business, or institution, formally or informally employed; (b) inactive: without productive activity or seeking employment, i.e., homemakers, students, and retirees; (c) unemployed: not working but seeking employment. This information was obtained through information from parents and, for stratified analysis purposes, categorized as non-unemployed (if a) or unemployed (if b or c).

- Family income: average earnings of the family over the preceding three months, categorized by minimum wages (MWs): up to 1.0 MW; 2.0–3.0 MW; 3.01–4.0 MW; >4.0 MW. This was evaluated by the respondent, considering the national minimum wage at the time of data collection, which was equivalent to EUR 35 [19]. For stratified analysis, this information was categorized as ≤1 MW; 2–3 MW; and ≥4 MW.
- Housing condition: A housing score adapted from Ludermir AB [20] was used, considering the components of the residence, to which a score was assigned. Thus, the wall was scored as 1 or 0 depending on whether it was made of brick or other materials; the roof was assigned a score of 1 and 0 (tile/slab and other materials); floor: 2—ceramic/wood, 1—cement, 0—dirt; water supply: 3—public network, 2—fountain, 1—well or spring, 0—other water supply forms; sewage system: 2—public network, 1—septic tank, 0—no sewage system. The arithmetic mean of the sum of each item was calculated, and individuals with a value below the mean were classified as having poor housing conditions; those with a value around the mean (mean ± 1 standard deviation (SD)) as medium, and those above the mean (+1 SD) as having good conditions. This was stratified in the analysis into two categories: not precarious (good and medium) and precarious.
- Household overcrowding: The overcrowding index proposed by Hill et al. [21] was used, considering three categories: 1—no overcrowding: fewer than four people per household and fewer than two people per room; 2—moderate overcrowding: fewer than four people per household and two or more people per room, or four or more people per household but fewer than two per room; 3—intense overcrowding: four or more people per household and at least two per room. For analysis, two categories were considered: no overcrowding and overcrowded (moderate/intense).
- Nutritional status: The weight-for-age anthropometric index was used, calculated at the time of inclusion in the study using the table recommended by the WHO [22]. For analysis, children with nutritional deficits were those who had two z-scores below the median value of the reference population in this index; and those without nutritional deficits were those who had two or more z-scores above the median reference value.
- Maternal education: Number of years of completed education. This was stratified into: level 1 (no formal education and/or unable to read or write, except for own name); level 2 (able to read and write and/or up to six years of schooling); level 3 (seven to 12 years of schooling); level 4 (>12 years of schooling).
- Frequent alcohol use was considered the consumption of at least one alcoholic drink, equivalent to 10 or 12 g of alcohol [23], per week during pregnancy.
- Birth spacing: the minimum interval between the birth of the child under study and the mother’s preceding or subsequent pregnancy, reclassified in accordance with WHO guidelines [24] as: 1. Less than 2 years; 2. 2 years or more.
- Breastfeeding Status: This variable pertains to whether a child is receiving maternal breast milk at the time of data collection. Operationalization divides children into two categories: “Yes” denotes those still breastfeeding at 2 years of age, while “No” signifies those who have ceased breastfeeding by this age.
- Breastfeeding Duration: This variable quantifies the total duration of breastfeeding, including both exclusive and non-exclusive breastfeeding, measured in months. It is operationalized as follows: “≤23 months” for children breastfed for 23 months or less, and “≥24 months” for those breastfed for 24 months or more.
- Number of prenatal visits: According to WHO guidelines [25], which recommend a minimum of four prenatal visits.

### 2.5. Data Analysis Strategy: Hierarchical Modeling

**Figure 2:**
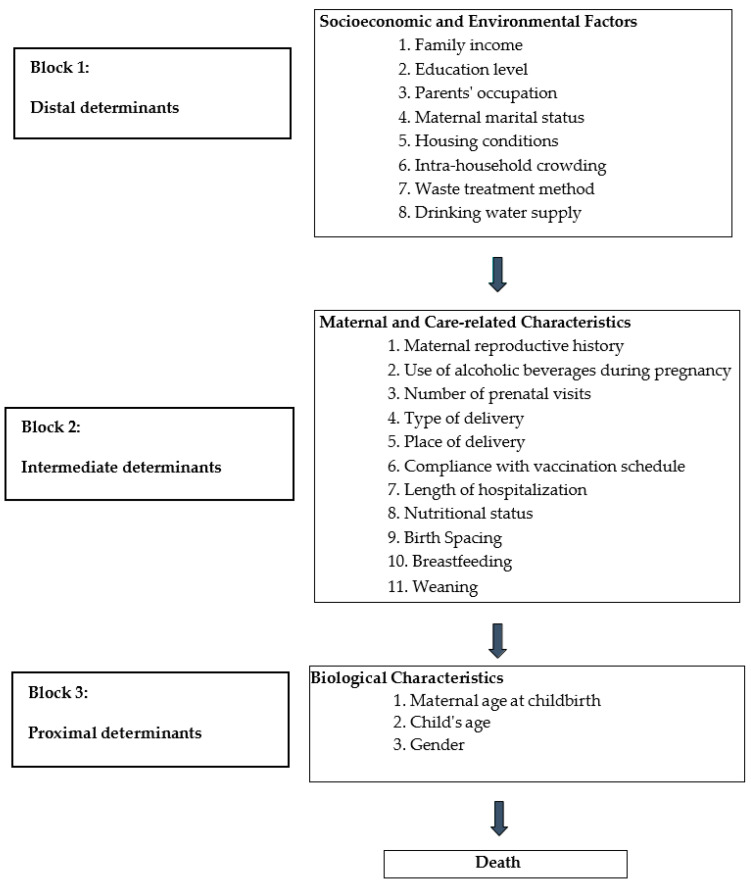
Explanatory Model for Under-Five Mortality. Adapted from Mosley Chen [15].

From an operational standpoint, the predictive factors under investigation were grouped into three hierarchical levels, each corresponding to a block of variables, according to the conceptual model proposed by Mosley and Chen [15] (Figure 2).

### 2.6. Statistical Analysis

The data analysis was conducted in two stages:

1. Initial descriptive analysis:

Quantitative variables were reported as mean ± SD or median with interquartile range if they did not follow a normal distribution. Categorical variables were described using absolute and relative frequencies.

The chi-square test for trend was used to study ordinal categorical variables. The effect size of explanatory variables was estimated by calculating the odds ratio (OR), with a 95% confidence interval (95% CI).

2. Binary logistic regression analysis, hierarchical method.

Categorical and discrete variables were kept as such, adopting cutoff points used in the literature. The hierarchical theoretical framework was used as a reference for building a model that allowed adjustment for confounding factors (see Figure 2). Beforehand, the absence of multicollinearity among explanatory variables was verified, as well as the goodness-of-fit of the data through the Hosmer–Lemeshow test.

The proposed model discriminated hierarchically higher predictive factors (distal) as exerting their action on those situated lower (intermediate and proximal) [26].

Since many factors under study could confound the associations, the variables were hierarchically organized into blocks for the selection of confounding factors most strongly associated with deaths in children under five years old.

Initially, variables with a *p*-value ≤ 0.20 in univariate analysis were selected. These variables were then analyzed together with the variables from the block to which they belonged in the conceptual model. Those that, in the intra-block analysis, had a *p*-value ≤ 0.10 were considered confounding factors and included as adjustment variables for the hierarchically lower variables according to the model. This procedure obviously relativizes the importance of the statistical significance of the distal factors by their successive insertion in the subsequent steps of the analysis regardless of losing statistical significance in this procedure.

In summary, according to the model, Block 1 of more distal variables, which will adjust all hierarchically lower variables, consists of variables that in univariate analysis had a *p*-value ≤ 0.20 and in analysis with other variables in the same block had a *p*-value ≤ 0.10. These variables from the more distal block were used to adjust those from subsequent blocks, in which the process and criteria for statistical significance were the same.

Binary logistic regression analysis and the hierarchical method from the SPSS 26.0 software for Windows (SPSS Inc., Chicago, IL, USA) were used with initial model saturation by including all variables from each block that had a univariate analysis *p*-value ≤ 0.20. Then, the variables from the block that had a *p*-value ≤ 0.10 after adjustment by other variables in the block were adjusted by hierarchically higher variables until only those within each block had a *p*-value < 0.10.

## 3. Results

*Sample Characterization*

A total of 1020 children, 340 cases, and 680 controls were recruited for this study, with a slight predominance of males (55.9%). The median weight of the children at admission and respective interquartile range (IQR) was 7600 g (5000), and the median (IQR) age was 15 (30) months. The mean (SD) maternal age was 24.6 (6) years, with the majority having less than six years of schooling (62.2%). A total of 44.7% of the deaths occurred within the first year. The median duration of breastfeeding was 21 months, with an interquartile range (IQR) of 7 months. The primary causes of death (cases) and hospitalization (controls) are presented in Table 1. The sample characterization is further detailed in Table 2, Table 3 and Table 4.

Respiratory infections topped the list of hospitalizations (31.6%), followed by malnutrition (20.2%) and malaria (15.3%). The leading cause of death was malaria (21.8%). A total of 76.1% of deaths occurred within the first 24 h of admission.

In the bivariate analysis, among distal determinants, maternal education revealed a statistically significant difference concerning the occurrence of death, with mothers without education or with low education having 10 times higher odds of having children who died before the age of five. Similarly, increased odds of death were observed with moderate to intense intrahousehold crowding, unemployed parents or caregivers, inappropriate household waste disposal methods, and consumption of water from inappropriate sources, as well as mothers who do not cohabit with their partners. The values of the socioeconomic and environmental variables found in this analysis are presented in Table 2.

Among intermediate determinants, statistically significant factors include alcohol consumption during pregnancy and hospitalization time ≤24 h. The odds of death in children under five were 3.5 times higher in mothers who consumed alcoholic beverages during pregnancy and eight times higher within the first 24 h of hospitalization. Poor nutritional status, incomplete vaccination schedules for the child’s age, and short interbirth intervals were also associated with death occurrence, as observed in Table 3.

Among proximal determinants, only maternal age (≤19 years and ≥35 years) was associated with death in children under five, showing twice the odds of death in children under five when compared to mothers aged 20 to 34 years (Table 4).

The hierarchical analysis revealed that the final model (stage 3) containing the variables caregiver’s education level, maternal marital status, caregiver’s occupation, alcohol consumption during pregnancy, type of delivery, length of hospital stay, nutritional status, interbirth interval, and maternal age at the time of pregnancy was statistically significant in predicting deaths in this group of children [X2 (12) = 191.584; *p* < 0.001. R2 Nagelkerke = 0.375]. Table 5 presents the final model of binary logistic regression analysis using the hierarchical method.

## 4. Discussion

Reducing mortality in children under five to lower than 25 per 1000 live births is one of the sustainable development goals related to child health [3]. The study assessed the influence of distal, intermediate, and proximal determinants on hospital deaths in children under five at David Bernardino Pediatric Hospital, a reference in pediatric care in Angola. The most relevant findings reveal that the majority of deaths occurred due to preventable causes, with an emphasis on distal and intermediate determinants.

Among the factors associated with under-five mortality in developing countries, socioeconomic factors are pointed out as deterministic relative to environmental factors, maternal characteristics, healthcare, nutritional status, and pre-existing health conditions [16,17,27].

Hierarchical modeling was chosen in this study as an alternative to traditional analysis methods due to the advantage of considering both “biological” and “statistical” aspects, structuring the investigation of risk factors and facilitating the interpretation of results [27,28]. The hierarchy of independent variables established in the conceptual framework [27] was maintained throughout the data analysis, allowing for the selection of those most strongly associated with the outcome of interest.

In this study, malaria was the leading cause of death followed by acute respiratory diseases, malnutrition, and gastrointestinal diseases. Approximately 35% of the demand for curative care in Angola is attributed to malaria, a pathology responsible for 20% of hospital admissions and 40% of perinatal deaths [6]. These findings are supported by previous studies conducted in Angola [9,10,29] and other African countries [30,31]. It is possible that the results are related to the high endemicity of the disease in the country, where it remains a significant public health problem, compounded by a lack of access to quality healthcare services associated with precarious socioeconomic conditions.

Poor environmental sanitation and failure to comply with basic prevention measures such as water treatment and food safety, as described in the country’s Multiple Indicator Cluster Survey [6], also contribute to the high prevalence of both acute diarrheal diseases and respiratory diseases. These findings underscore the need for improvements in environmental conditions, expanded access to healthcare services, and greater effectiveness in case management.

Among the distal (socioeconomic) factors, maternal education level was of great importance in predicting deaths in children under five, both in univariate and hierarchical analysis, showing a higher likelihood of deaths in children under five born to mothers with no education or low education compared to those with more than 12 years of schooling. Several studies in Africa have described a significant association between low maternal education and deaths in children under five [32,33,34,35,36]. The relevance of this data may be due to the fact that maternal education is considered an important marker of the mother’s and family’s socioeconomic status, playing a significant role in adopting healthy behaviors and habits with a direct impact on the health of their children [33,35].

Although some studies [4,36] have shown an association between unemployment, family income, and higher mortality in children under five, in this study, these variables did not show statistical significance even in univariate analysis. This could be due to the culturally sensitive nature and complex approach of this information, as most surveyed mothers were unaware of their partners’ monthly income, and it is very likely that the information provided by the mother does not reflect reality in the same way. Additionally, unemployed caregivers also did not have higher chances of having children who died before the age of five.

Alcohol consumption during pregnancy has been associated with mortality in children under five and various other health problems [37,38,39]. In this study, the odds of death were 3.8 times higher in mothers who had frequent alcohol habits during pregnancy. The consumption of alcoholic beverages and other legal or illegal drugs can directly affect fetal development; it can alter placental function, reducing the exchange of oxygen and nutrients between the fetus and the mother [39]. This is likely to increase the risk of childhood death due to the negative impact on the mother’s nutritional status during pregnancy and considering the biological relationship between the mother and fetus during this period, where the latter is extremely dependent on the maternal organism for its development.

The odds of death were 13.7 times higher in children with less than 24 h of hospitalization, highlighting the weight of epidemiological factors independent of the hospital in child mortality. This result aligns with findings from previous studies in the same hospital [40] and underscores the need to strengthen primary healthcare strategies for populations and improve the referral and counter-referral system. On the other hand, the findings may be justified by the late arrival of children at the hospital, often in advanced stages of illness, either directly from home or from alternative treatment institutions.

Children with moderate to severe malnutrition were twice as likely to die during hospitalization compared to those without malnutrition, even after adjusting for all other variables; findings that coincide with others described in the literature [10,11,12,13,14,15,16,17,18,19,20,21,22,23,24,25,26,27,28,29,30]. Indeed, malnourished children have a deficient immune response, with more severe infections than well-nourished children; thus, measures aimed at preventing low birth weight and maintaining adequate nutritional status, provided through proper prenatal care and follow-up in childcare consultations, are interventions that can significantly reduce the risk of hospitalization and death in this age group.

Short interbirth spacing presented, in this study, a chance of about twice as high in predicting deaths in children under five. The WHO identifies the period between two consecutive live births as a critical determinant of childhood mortality risks and recommends a spacing of three to five years between two consecutive births to reduce risks to the health of children and mothers [24]. Short intervals between births are described in the literature as important predictors of adverse outcomes for children, especially in developing countries [35,36,40], and maternal depletion, infection transmission, and sibling competition may be some of the main explanations for this association [41,42,43,44].

In summary, shorter birth intervals do not allow women to fully recover their physical well-being and the ideal distribution of energy and micronutrients from the previous pregnancy, which may result in suboptimal fetal development and an increased risk of mortality for the child born after the short interval [44,45]. Short intervals between births predispose the younger child to a set of diseases similar to those of the older sibling, compromising the development of the immune system, which for certain communicable diseases, such as measles, tends to have significantly higher fatality rates [44]. Finally, because developing countries typically have a large number of extended and poor families, children with close spacing are more likely to compete for the same family resources, especially for breast milk, a fundamental food in the first years of life for the index child [45,46,47,48].

The relationship between breastfeeding duration and child mortality under five years of age is well-established in public health. In this study, the duration of breastfeeding showed significant associations with mortality risk. Children breastfed for 23 months or less exhibited higher odds of mortality (OR 1.5, 95% CI: 1.0–2.2) compared to those breastfed for 24 months or more. These findings are consistent with the current scientific literature [47,48,49] and guidelines from the World Health Organization (WHO), emphasizing the critical role of breastfeeding in reducing child mortality [1].

Breastfeeding provides essential nutrients and protective factors that strengthen the immune system, thereby reducing the incidence and severity of infections such as respiratory illnesses and diarrhea, which are major contributors to child mortality worldwide [1]. Moreover, prolonged breastfeeding is associated with long-term health benefits, including lower rates of chronic diseases and improved cognitive development [49]. Promoting breastfeeding practices aligns with WHO recommendations, which advocate exclusive breastfeeding for the first six months of life followed by continued breastfeeding for up to two years or beyond [1], and is crucial for improving child survival rates globally.

Higher mortality in children under five among live births of mothers at extreme ages, (aged ≤19 years and those over 35 years) is suggested in several studies [50,51,52,53,54,55,56,57]. In this study, maternal age was found to be associated with this outcome in both univariate analysis and hierarchical logistic regression; the findings demonstrate the need for reinforcement in family planning efforts and the strengthening of primary healthcare. However, in some studies, this association is not sufficiently clear as the variable may be influenced by various factors, such as the quality of access to healthcare services, socioeconomic conditions, and education [54].

Several physiological, cultural, and socioeconomic factors may contribute to this unfavorable outcome. Physiologically, the younger the mother’s body, the higher the likelihood of pregnancy and childbirth complications that increase the risk of mortality in children under five [50]. Socioculturally, adolescent mothers are more likely to face stigma and barriers in accessing maternal health services, predisposing children to a higher risk of death [35,57] Socioeconomically, compared to adults, adolescent mothers are more likely to have low levels of education and unfavorable economic conditions, which have been considered predictors of mortality in children under five [4,35,37].

On the other hand, advanced maternal age is associated with increased stillbirths, premature births, and intrauterine growth restriction, as well as chromosomal abnormalities resulting from multiple factors. In some contexts, it is women with lower socioeconomic status, lower education levels, and higher parity, while in others, it is educated women who delayed pregnancy for career reasons. Older mothers are at higher risk of obesity, diabetes, hypertension, and pregnancy-related complications [54,57].

The lack of association between socioeconomic factors—family income, household overcrowding, housing conditions, and unemployment—and higher mortality in children under 5 years old in this study suggests that other determinants may be more relevant. However, it is necessary to consider that these factors may indirectly influence child health through access to and quality of healthcare services, and therefore, the lack of direct association does not imply that these factors are irrelevant. Additionally, the study only analyzed hospital mortality, excluding possible impacts on deaths occurring outside the hospital environment. The sample size may have affected the results, highlighting the need for additional studies, including those with longitudinal designs covering different levels of healthcare, to achieve a more comprehensive understanding of determinants of mortality in children under five in Angola and to guide more effective public policies.

Another limitation of this study is the restriction of data collection to weekdays and business hours (8:00 AM to 4:00 PM). Cases of mortality occurring after 4:00 PM and on weekends were automatically excluded due to logistical constraints in interviewing caregivers. This exclusion could introduce bias by omitting deaths that may occur during non-standard hours, potentially compromising the study’s ability to fully represent the spectrum of infant mortality during the study period. It is crucial to consider this limitation when interpreting the findings, as it could affect this study’s generalizability and its conclusions regarding under-five mortality patterns.

## 5. Conclusions

Based on the main findings of this study, low maternal education, poor nutrition, and limited emergency healthcare access are significant distal and intermediate determinants contributing to high hospital mortality rates among children under five in Angola. Addressing these issues through targeted public policies is crucial for improving child health outcomes. Additionally, efforts to reduce social inequalities and enhance access to healthcare services are essential to achieve the Sustainable Development Goal targets for 2030. Angola must prioritize investments in primary healthcare quality and accessibility, particularly for women and children, to ensure sustainable improvements in child health.

## Figures and Tables

**Table 1:** Frequency distribution of diagnoses for cases (cause of death) and controls (hospital discharge). David Bernardino Pediatric Hospital (Angola): 1 May 2022–30 June 2023.

ICD-10	Diagnosis	Discharge Status				Total	
		Control		Case			
		No	%	No	%	No	%
R10.0	Acute abdomen	5	0.7	5	1.5	10	1.0
D64.9	Severe anemia	23	3.4	21	6.2	44	4.3
Q20–Q26	Congenital heart disease	20	2.9	10	2.9	30	2.9
E43	Severe unspecified protein-energy malnutrition	137	20.1	50	14.7	187	18.3
A09	Diarrhea and Presumed Infectious Gastroenteritis	53	7.8	32	9.4	85	8.3
G00-G09	Inflammatory diseases of the central nervous system	22	3.2	26	7.6	48	4.7
J00–J99	Acute Respiratory Infections	215	31.6	62	18.2	277	27.2
D57	Sickle cell disease	20	2.9	9	2.6	29	2.8
K92.2	Upper gastrointestinal bleeding	2	0.3	4	1.2	6	0.6
G91	Hydrocephalus	19	2.8	5	1.5	24	2.4
L08.9	Localized infection of skin and subcutaneous tissue	13	1.9	2	0.6	15	1.5
T18	Foreign body ingestion	8	1.2	-	-	8	0.8
B50.8	Unspecified malaria by *Plasmodium falciparum*	104	15.3	74	21.8	178	17.5
Q44	Congenital malformations of the gallbladder, bile ducts, and other unspecified liver diseases	1	0.1	4	1.2	5	0.5
C80	Malignant neoplasm, unspecified site	3	0.4	-	-	3	0.3
P20-P29;M069;E10;A35;G81.0;B05;K40; I27	Others: Hypoxia, Arthritis, Diabetes, Tetanus, Non-specific flaccid paralysis, AIDS, Measles, Hernia, and Pulmonary Hypertension	15	2.2	10	2.9	25	2.5
P07	Prematurity	-	-	6	1.8	6	0.6
A41.9	Unspecified sepsis	7	1.0	13	3.8	20	2.0
S00–S09	Injuries to the head	13	1.9	7	2.1	20	2.0
	Total	680	100.0	340	100.0	1020	100.0

**Table 2:** Association between socioeconomic and environmental factors and the occurrence of deaths in children under five years old. David Bernardino Pediatric Hospital (Angola): 1 May 2022–30 June 2023.

Level 1	Case: n (%)	Control: n (%)	OR (CI 95%)
Maternal education			
No schooling	97 (44.7)	120 (55.3)	10.1 (4.5–22.8)
≤6 years	157 (38.2)	254 (61.8)	7.7 (3.5–17.1)
7–12 years	79 (27.5)	208 (72.5)	4.7 (2.1–10.6)
>12 years	7 (7.4)	87 (92.6)	1.0
Maternal marital status			
Single	158 (37.4)	264 (62.6)	1.4 (1.1–1.8)
With partner	182 (30.4)	416 (69.6)	1.00
Housing conditions			
Precarious	136 (35.5)	247 (64.5)	1.2 (0.9–1.5)
Non-precarious	204 (32.0)	433 (68.0)	1.00
Family income			
Unknown	493 (66.5)	248 (33.5)	2.2 (1.4–3.6)
≤1 MW	20 (62.5)	12 (37.5)	1.7 (0.7–3.9)
1.01–3 MW	107 (66.0)	55 (34.0)	2.1 (1.3–3.7)
≥3.01 MW	60 (70.6)	25 (29.4)	1.00
Intra-household crowding			
Moderate to intense crowding	701 (74.7)	237 (25.3)	2.1 (1.3–3.3)
No crowding	48 (58.5)	34 (41.5)	1.00
Caregiver’s integration into the workforce			
Unemployed	137 (30.6)	311 (69.4)	0.8 (0.6–1.0)
Not unemployed	203 (35.5)	369 (64.5)	1.00
Household waste collection and treatment			
Inappropriate	180 (30.3)	415 (69.7)	0.7 (0.6–0.9)
Appropriate	160 (37.6)	265 (62.4)	1.00
Source of drinking water			
Not appropriate	324 (34.5)	614 (65.5)	2.2 (1.2–3.8)
Appropriate	16 (19.5)	66 (80.5)	1.00

**Table 3:** Association between maternal and care-related characteristics and the occurrence of deaths in children under five years old. David Bernardino Pediatric Hospital (Angola): 1 May 2022–30 June 2023.

Level 2	Cases: n (%)	Controls: n (%)	OR (CI 95%)
Parity			
≥3	151 (33.1)	305 (66.9)	1.0 (0.8–1.3)
≤2	189 (33.5)	375 (66.5)	1.00
Alcohol consumption during pregnancy			
Yes	222 (48.5)	236 (51.5)	3.5 (2.7–4.7)
No	118 (21.0)	444 (79.0)	1.00
Number of prenatal visits			
≤3	219 (33.7)	430 (66.3)	1.1 (0.8–1.4)
≥4	121 (32.6)	250 (67.4)	1.00
Type of delivery			
Cesarean	13 (19.1)	55 (80.9)	0.5 (0.2–0.8)
Vaginal	327 (34.3)	625 (65.7)	1.00
Place of delivery			
Non-Hospital	106 (38.0)	173 (62.0)	1.3 (1.0–1.8)
Hospital	234 (31.6)	507 (68.4)	1.00
Length of hospital stay:			
≤24 h	70 (76.1)	22 (23.9)	7.8 (4.7–12.8)
>24 h	270 (29.1)	658 (70.9)	1.00
Nutritional status			
With nutritional deficit	202 (39.1)	314 (60.9)	1.7 (1.3–2.2)
No nutritional deficit	138 (27.4)	366 (72.6)	1.00
Vaccination schedule			
Incomplete for age	88 (39.5)	135 (60.5)	1.4 (1.0–1.9)
Complete for age	252 (31.6)	545 (68.4)	1.00
Birth Spacing *			
<24 months	113 (39.9)	170 (60.1)	2.0 (1.4–2.8)
≥24 months	85 (25.2)	252 (74.8)	1.00
Breastfeeding status at 2 years of age			
No	42 (37.7)	79 (65.3)	0.3 (0.2–0.4)
Yes	375 (67.0)	185 (33.0)	1.00
Breastfeeding duration **			
≤23 months	112 (36.4)	196 (63.6)	1.5 (1.0–2.2)
≥24 months	53 (27.3)	141 (72.7)	1.00

* Children born to primiparous mothers were excluded from the analysis; ** Data collected only for weaned children at the time of data collection.

**Table 4:** Association between biological factors (maternal and child) and the occurrence of deaths in children under five years old. David Bernardino Pediatric Hospital (Angola): 1 May 2022–30 June 2023.

Level 3	Case: n (%)	Control: n (%)	OR (CI95%)
Gender			
Male	190 (33.3)	380 (66.7)	1.0 (0.8–1.3)
Female	150 (33.7)	300 (66.7)	1.00
Child’s age			
≤12 months	152 (33.3)	305 (66.7)	1.0 (0.8–1.3)
>12 months	188 (33.4)	375 (66.6)	1.00
Maternal age at delivery			
10–19 Years	142 (46.0)	167 (54.0)	2.5 (1.9–3.4)
20–34 Years	152 (25.3)	449 (74.7)	1.00
≥35 Years	46 (41.8)	64 (58.2)	2.1 (1.4–3.2)

**Table 5:** Final model of hierarchical binary logistic regression analysis of risk factors for deaths in children under five years old: David Bernardino Pediatric Hospital (Angola): 1 May 2022–30 June 2023.

Factors	Β	S.E	Wald	Df	Sig.	OR (CI 95%)
Caregiver’s occupation (unemployed)	−0.8	0.3	10.1	1	0.001	0.4 (0.3–0.7)
Marital status (without a partner)	−0.1	0.3	0.3	1	0.580	0.9 (0.5–1.4)
Maternal education						
No education	1.5	0.7	4.9	1	0.027	4.3 (1.2–15.7)
≤6 Years	1.2	0.7	3.5	1	0.060	3.4 (1.0–12.1)
7–12 Years	1.1	0.7	2.9	1	0.087	3.1 (0.9–11.5)
>12 Years			5.4	3	0.144	
Frequent alcohol consumption during pregnancy (Yes)	1.4	0.2	38.0	1	0.001	3.8 (2.5–5.9)
Type of delivery (cesarean section)	−1.4	0.8	3.3	1	0.071	0.3 (0.1–1.1)
Length of hospital stay (≤24 h)	2.6	0.4	40.7	1	0.001	13.8 (6.2–30.8)
Nutritional status (with deficit)	0.8	0.2	12.3	1	0.001	2.1 (1.4–3.2)
Birth Spacing (<24 months)	0.5	0.2	6.1	1	0.014	1.7 (1.1–2.5)
Breastfeeding duration (<2 years)	0.42	0.20	4.00	1	0.045	1.5 (1.1–2.1)
Maternal age at the time of delivery						
≤19 Years	1.7	0.3	27.4	1	0.001	5.6 (3.0–10.8)
20 to 34 Years			30.5	2	0.001	
≥35 Years	0.8	0.3	7.4	1	0.006	2.1 (1.2–3.7)
Constant	−3.5	0.7	28.4	1	0.001	0.03

Model 1 = caregiver’s education + marital status + caregiver’s occupation; Model 2 = Model 1 + alcohol consumption during pregnancy + type of delivery + length of hospital stay + nutritional status + interpregnancy interval (Birth Spacing) + Breastfeeding duration; Model 3 = Model 2 + maternal age at time of pregnancy. β = Coefficient; Standard error (SE); df = degrees of freedom; sig. = Statistical significance; OR Odds ratio; CI = confidence interval.

## Data Availability

The data used in this study belong to DBPH and are under the auspices of IHMT. They may be available upon request to researchers who meet the criteria for accessing confidential data. Data requests should be directed to Professor Luís Varandas, Supervisor of the lead researcher of this study and a Professor at IHMT, whose email address is varandas@ihmt.unl.pt. Requestors should provide details about the purpose of the request and must meet the requirements of the Scientific Council of IHMT. Data should only be used for the purpose defined in the request.

## Supplementary Materials

The Data Collection Instrument can be downloaded at: https://www.mdpi.com/article/10.3390/ijerph21081062/s1. Supplementary File: Data Collection Instruments.
