# Supplementary material for: Under-Five Mortality and Associated Risk Factors in Children Hospitalized at David Bernardino Pediatric Hospital (DBPH), Angola: A Hierarchical Approach
Source: Int J Environ Res Public Health. 2024 Aug 14;21(8):1062. doi: 10.3390/ijerph21081062 (PMC11354039; doi:10.3390/ijerph21081062)
Supplement: Supplementary file 1 [file ijerph-21-01062-s001.zip › ijerph-3054089-supplementary.pdf]

Under-five mortality and associated risk factors in children hospitalized at David Bernardino Pediatric Hospital (DBPH), Angola.

Israel Avelino, Joaquim Van-Dúnem, Luís varandas

ANNEXES

Data Collection Instruments

Mortality and Associated Factors in Children under Five Years of Age Hospitalized at David Bernardino Pediatric Hospital from May 1, 2022, to June 30, 2023

(Questionnaire)

Record No.__________________________Clinical File No.__________________________

Researcher:_______________________Data Collection Date: _____/_____/________

Interviewee:______________________________

(Relationship)________________________

Phone:__________________

A. CHILD IDENTIFICATION

1. Date of birth: _____/_____/____
2. Age: ________________(years/months)
3. Date of hospitalization: _____/_____/_____
4. Admission diagnosis: ____
5. Place of origin: (Neighborhood/municipality) _______________________
6. Province: _____________________________
7. Referral guide from another health unit: a) Yes (__); b) No (__)

B. BIOLOGICAL AND CLINICAL CHARACTERISTICS OF THE CHILD

1. Gender: a) Male (__) b) Female (__)
2. Does the child have an infant health card? a) Yes (__) - verify the card; b) No (__)
3. Was the child breastfed? a) Yes (__) b) No (__)

3.1. If yes: Is the child still breastfeeding? a) Yes (__) b) No (__)

4. Does the child consume any food other than breast milk? a) Yes (__) b) No (__).

4.1. If yes: what foods?____

5. What age was the child when started giving other foods?________________________
6. If already weaned, what age was the child when stopped breastfeeding?__________________
7. Does the child have a vaccination card?

a) Yes (__) (seen)

b) Yes (__) (not seen)

c) Had, but lost (__)

d) Never had (__)

8. Verification of vaccination card:

8.1. Compliance with the vaccination schedule for age: a) Yes (__) b) No (__)

8.2. Source of information:

a) Card (__)

b) Mother (__)

c) Pentavalent scar (injection in the buttock) (__)

d) Sabin (drop in the mouth) (__)

e) Measles (interscapular) (__)

f) BCG (scar on the arm) (__)

10. Has the child been hospitalized (for more than 24 hours) in the last twelve months?

a) Yes (__)
b) No (__)
c) IF YES, what was the cause?____
11. Anthropometry (physical examination)
a) Birth weight ____ g (verify infant health card) a) Don't know (__)
b) Entry weight: _______ kg
c) Length/height: ______cm
d) Head circumference _________cm
e) Nutritional status_____________________

C. MATERNAL CHARACTERISTICS

1. How old are you? (if someone other than the child's mother is responding, ask for the mother's age) __________________________
2. How old was the mother when she conceived this child?_______________ years
3. How many children has the mother had before this one?__________
4. Were they all born alive? a) Yes (__) b) No (__)
a) Born alive _____
b) Stillborn (≤28 weeks gestation or 1,000 g).______________
5. What is the birth date of the sibling born before this one?
____/____/____ or how old are they? ________.
6. Is he/she in good health? _________________________
7. Does the mother have children who have died? a) Yes (__) b) No (__)
7.1. If yes, record the child's age and cause of death
____
8. During the pregnancy of "Child", did Mrs. "Mother" smoke cigarettes?
a) Never (__)
b) Occasionally (< 1 per day) (__)
c) Frequently (At least 1 cigarette daily) (__)
d) Don't know / Not disclosed (__)

9. During the pregnancy of "Child", did Mrs. "Mother" consume any alcoholic beverages?
a) Never (__)
b) Occasionally (< 1 drink per week) (__)
c) Frequently (1 drink or more per week) (__)
d) Don't know / Not disclosed (__)

10. During the pregnancy of "Child", did Mrs. "Mother" use drugs? (Marijuana, crack, cocaine, etc.)
a) Never (__)
b) Occasionally (< 1 time per month) (__)
c) Frequently (1 time or more per week) (__)
d) Don't know / Not disclosed (__)

D. MATERNAL AND CHILD HEALTH CARE

1. During the pregnancy of "Child", did Mrs. "Mother" have any prenatal visits?
a) Yes (__), a.1) How many? (__)

b) No (__)

c) Don't know / Not disclosed (__)

2. In which month of pregnancy did Mrs. "Mother" have the first prenatal visit?

a) 1st Month (__); 2nd Month (__); 3rd Month(__); after the 3rd month (__)

b) Did not have prenatal care (__)

c) Don't know / Not disclosed (__)

3. Gestation period (duration of pregnancy)__________(months/weeks)

4. Place of birth: Hospital/maternity (__); at home (__); Other(__)

5. What was the type of delivery for "Child"?

a) Vaginal (__); Cesarean (__); Don't know / Not disclosed (__)

6. HIV infection: a) Yes (__); b) No (__)

6.1. If positive, is treatment being undertaken? a) Yes (__) b) No (__)

6.2. Is the child (hospitalized) currently breastfed? a) yes____________b) no______________. If weaned, for how long did breastfeeding last (months)?________________

7. What other health problems did Mrs. "Mother" have during pregnancy? (Spontaneous response)

____

____

____

____

E. SOCIOECONOMIC AND ENVIRONMENTAL DETERMINANTS

1. Can you read and write? a) Yes (__); b) No (__)

1.1. IF YES, how many years of schooling have you completed? __________________________

2. Do you have a job that earns money or provides food for the household?

a) Yes (__); b) No (__)

2.1. IF YES, what is the job? __________________________________

3. Average family income per month (EUR)

* ***Considering the national minimum wage at the time of data collection, in the local currency (kwanza), equivalent to 35 euros.***

a) (≤ 1.0 MW (__)

b) (2.0-3.0 MW (__)

c) (3.01-4.0 MW (__)

d) (>4.0 MW (__)

e) Prefer not to disclose (__)

4. Conditions of the child's residence:

4.1. Does the house where the child lives have electricity? a) Yes (__); b) No (__)

4.2. Building materials of the housing:

4.2.1. Roof: a) Tile/slab: (__); b) Other: (__)

4.2.2. Walls: a) Brick (__) b) Other: (__)

4.2.3. Floor: a) Wood/ceramic b) cement: (__) c) earth (__)

5. How many people live in the house where the child lives? (__)

5.1. How many people, on average, sleep in each room? (__)

5.2. Father lives at home? a) Yes (__); b) No (__)

5.3. Mother lives at home: a) Yes (__); b) No (__)

5.4. Siblings __________ (number)

5.5. Others _________ (number)
6. At the time of the child's birth, what was the water supply like in the home where Mrs. "Mother" lived? (Consider only the predominant type)
a) General network (__);
b) Well or spring on the property (__);
c) Fountain, well, or spring outside the property (__)
d) Non-existent (__)
e) Other (__)
7. At the time of the child's birth, what type of water was used for drinking in the home where Mrs. "Mother" lived?
a) Mineral water (__)
b) Filtered water (__)
c) Boiled and filtered water (__)
d) Tap water (__)
e) Other (__)
f) Don't know / Not disclosed (__)
8. Does the house have a toilet (latrine)?
a) Residence or yard (__)
b) Community (__)
c) Non-existent (__)
9. At the time of the child's birth, what was the type of drainage for the bathroom where Mrs. "Mother" lived?
a) General network (__)
b) Septic tank (__)
c) Other (__)
d) There was no bathroom (__)
e) Don't know / Not disclosed (__)
10. At the time of the child's birth, what was the waste collection method in the home where Mrs. "Mother" lived? (Consider only the predominant type)
a) Public service (__)
b) Burned / buried on the property (__)
c) Dumped elsewhere (not intended for that purpose) (__)
d) Other (__)
e) Don't know / Not disclosed (__)

F. EXIT CHARACTERISTICS

1. Date of Discharge: _____/_____/_____
2. Time: _____,_____
3. Length of hospital stay __________________Days
4. Condition at discharge:
a) Deceased (__); b) Home discharge (__); c) Transfer (__)
5. In case of death: on which day of the week did the death occur?
a) Monday to Friday (__);
b) Saturday or Sunday (__);
c) Holiday (__);
6. Was the diagnosis of the child's death confirmed by: Complementary examination?
a) Yes (__); b) No (__); c) Don't know / Not disclosed (__)

7. Surgery?

a) Yes (__); b) No (__); c) Don't know / Not disclosed (__)

8. Causes of death / approximate time between onset of illness and death / ICD (International Classification of Diseases. Each disease has a code given by letters and numbers) (Basic cause)

a) ICD:

b) ICD:

c) ICD:

9. Data collection closure date: _____/_____/_____

Interviewer:

____
